# Macrophage Membrane-Coated Nanoparticles for Immunomodulation and Bone Regeneration: Emerging Applications in Oral and Dental Implant Therapy

**DOI:** 10.3390/biomimetics11070482

**Published:** 2026-07-10

**Authors:** Sara Derhambakhsh, Tulio Fernandez-Medina, Elsa Antunes, Suchandan Sikder, Ernest Jennings, Catherine M. Miller

**Affiliations:** 1 College of Medicine and Dentistry, James Cook University, Cairns, QLD 4878, Australia; 2 School of Dentistry, The University of Queensland, Brisbane, QLD 4006, Australia; 3 College of Science and Engineering, James Cook University, Townsville, QLD 4811, Australia; 4 Australian Institute of Tropical Health and Medicine, James Cook University, Cairns, QLD 4878, Australia

**Keywords:** macrophage membrane-coated nanoparticles, immune modulation, biomimetic nanocarriers, inflammation, tissue regeneration, oral diseases, dental implants

## Abstract

Macrophage membrane-coated nanoparticles (MMNPs) are an emerging class of biomimetic nanoplatforms that combine the immune-regulatory functions of macrophages with the structural versatility of synthetic nanoparticles (NPs). By retaining key membrane proteins and receptors, MMNPs exhibit natural targeting capabilities, immune interactions, and inflammatory site homing, making them promising tools for immunomodulation and targeted therapy. This review summarizes macrophage biology relevant to immune regulation and discusses how nanoparticle core properties, including size, surface charge, composition, and mechanical characteristics, influence membrane coating efficiency, stability, and biological performance. Current fabrication and characterization strategies for MMNPs are also discussed. Particular emphasis is placed on the therapeutic applications of MMNPs in inflammatory disorders, tissue regeneration, and oral and dental implant-related applications. Recent studies demonstrate that MMNPs can modulate macrophage polarization, sequester pro-inflammatory cytokines, remodel the immune microenvironment, and promote tissue repair and bone regeneration, highlighting their potential to improve implant integration and reduce inflammation-associated implant failure. Despite these promising advances, challenges remain regarding large-scale manufacturing, membrane preservation, reproducibility, and long-term biosafety. Continued interdisciplinary research in nanotechnology, immunology, and biomaterials engineering is expected to accelerate the clinical translation of MMNPs for regenerative and immunomodulatory therapies.

## 1. Introduction

Inflammation underlies a wide range of pathological conditions, including autoimmune diseases, cancer, chronic inflammatory disorders, and complications associated with implanted biomaterials. While acute inflammation is essential for host defence and tissue repair, dysregulated or persistent inflammatory responses may lead to tissue damage and disease progression [1]. Consequently, many therapeutic strategies aim to either suppress excessive immune activation or modulate immune responses to restore homeostasis rather than completely inhibiting immunity. Immune suppression typically involves dampening immune activity to prevent pathological tissue damage, whereas immune modulation encompasses a broader approach, seeking to recalibrate leukocyte behaviour and inflammatory signalling pathways [2]. For example, autoimmune diseases arise from inappropriate immune recognition of self-antigens, resulting in chronic inflammation [3,4], whereas in cancer, insufficient immune activation allows tumour cells to evade immune surveillance [5]. Understanding and controlling the balance between immune suppression and immune modulation is, therefore, central to the development of effective therapies across diverse clinical contexts.

**Figure 1:**
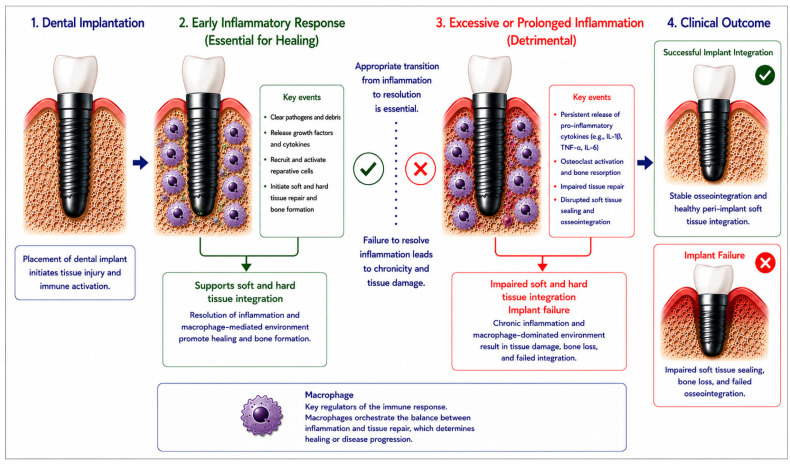
Schematic illustration of the host immune response following dental implant placement. Implant insertion initiates tissue injury and immune activation, triggering an early inflammatory response that is essential for successful healing. When inflammation is resolved appropriately, macrophages coordinate tissue repair by regulating cytokine production, recruiting reparative cells, and promoting soft and hard tissue integration, ultimately resulting in stable osseointegration and long-term implant success. In contrast, excessive or prolonged inflammation leads to persistent production of pro-inflammatory cytokines, impaired tissue repair, osteoclast activation, bone resorption, and disruption of osseointegration, culminating in peri-implant tissue breakdown and implant failure. The figure highlights the central role of macrophages in balancing inflammatory and regenerative responses during peri-implant healing, and it emphasizes their importance as therapeutic targets for immunomodulatory strategies to improve implant integration and long-term clinical outcomes.

Dental implants represent a clinically important application in which immune modulation plays a decisive role in treatment success. Following implantation, the host immune system initiates an inflammatory response that is essential for early healing; however, excessive or prolonged inflammation can impair osseointegration and lead to implant failure [6,7]. Macrophages are key regulators of this process, as their polarization state influences the balance between pro-inflammatory and regenerative responses. A timely transition from the pro-inflammatory M1 phenotype to the anti-inflammatory M2 phenotype is critical for resolving inflammation and promoting bone formation around the implant [8]. Therefore, strategies that can precisely modulate the local immune environment, particularly macrophage behaviour, have the potential to significantly enhance implant integration and long-term clinical outcomes (Figure 1).

However, achieving precise and context-dependent immune regulation remains a significant challenge, as conventional therapeutic approaches often lack spatial and temporal control over immune responses. In this regard, advanced material-based platforms have emerged as promising tools for the targeted and controlled modulation of immune activity. Nanoparticles (NPs), typically defined as materials with dimensions ranging from 1 to 100 nm (and in some cases extending to several hundred nanometres), have attracted considerable attention in biomedical applications as versatile carriers for biological and bioactive components [9]. Their high surface-area-to-volume ratio and tuneable physicochemical properties allow for precise control over parameters such as particle size, shape, surface chemistry, composition, mechanical stability, and degradation behaviour during fabrication. These features enable NPs to be rationally engineered for controlled interactions with cells and tissues [9]. By tailoring these properties, NPs can facilitate the stable presentation of biomolecular interfaces, localize biological functions to specific sites, and provide structural support for otherwise fragile biological materials. In the context of immune modulation, NPs are increasingly employed not as primary therapeutic agents, but as supportive scaffolds that enable the organization, stabilization, and spatial confinement of functional biological membranes.

In recent years, cell membrane-coated nanoparticles (CMCNPs) have been developed as a biomimetic strategy that cloaks synthetic NPs with natural cell membranes [10]. The membrane coating acts as an intermediary layer, isolating the nanoparticle core from the biological environment while regulating the exchange of molecules and signals. By preserving native membrane proteins, including self-recognition markers and immune-regulatory ligands, this approach enables immune evasion, prolonged circulation, and reduced immunogenicity. Combining the inherent biological functions of cell membranes with the properties of synthetic NPs, CMCNPs have emerged as a powerful platform for therapeutic applications, such as targeted drug delivery, vaccine development, detoxification, and immunomodulation [11,12,13].

**Figure 2:**
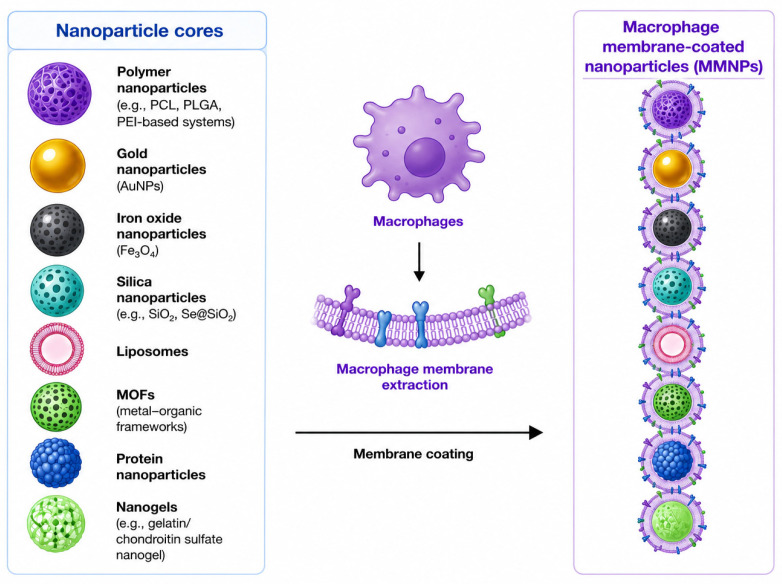
Schematic illustration of MMNPs consisting of various nanoparticle cores encapsulated within macrophage-derived membranes.

Membranes derived from various cell types, including erythrocytes, mesenchymal stem cells, platelets, cancer cells, and leukocytes, have been explored for this purpose [14,15,16,17,18]. Leukocytes possess distinctive characteristics that make them highly valuable for therapeutic and diagnostic applications. Their cell membranes display a diverse array of receptors, ligands, and signalling proteins that confer advantages such as immune evasion, prolonged circulation, trans-endothelial migration, and selective targeting of diseased or inflamed tissues. Among these, macrophage membranes are of particular interest due to the central role of macrophages in regulating inflammatory responses. Macrophage membrane-coated nanoparticles (MMNPs) exploit the immunomodulatory and anti-inflammatory properties of native macrophages, enabling biologically relevant interactions at inflammatory interfaces while minimizing undesired immune activation [19]. A wide range of nanoparticle core materials can be employed as supportive platforms in macrophage membrane-coated systems, including polymeric NPs (e.g., PLGA), inorganic NPs (gold, iron oxide, silica), soft carriers, such as liposomes and nanogels, and porous structures like metal–organic frameworks (Figure 2). In this strategy, the nanoparticle core primarily provides mechanical stability, surface area, and cargo-loading capacity, enabling the stable presentation of macrophage membranes while the membrane preserves functional surface proteins, such as chemokine receptors (e.g., CCR2), which support inflammation-associated targeting and immune signalling.

In this review, we first outline the role of macrophages in inflammation and immune regulation, followed by an overview of the immunomodulatory applications of macrophage membrane-coated nanocarriers. We then discuss how the physicochemical properties of nanoparticle core materials influence coating efficiency and biological performance. Finally, we summarize the therapeutic applications of MMNPs in inflammatory disorders, tissue regeneration, and oral diseases, with particular emphasis on bone healing and dental implant-related applications.

## 2. The Role of the Macrophage in the Inflammatory Response

**Figure 3:**
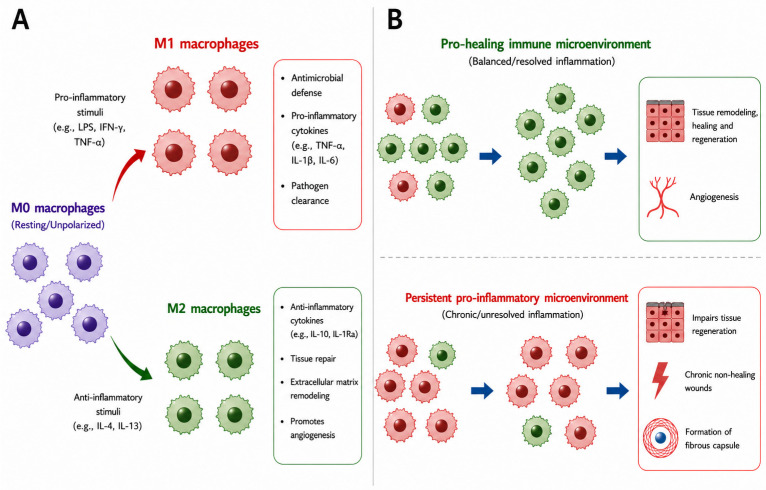
Macrophage polarization and tissue outcomes. (**A**) M0 macrophages can polarize into pro-inflammatory M1 macrophages, promoting immune defence, or anti-inflammatory M2 macrophages, and supporting tissue repair. (**B**) The balance of M1 and M2 macrophages determines tissue fate: M2 dominance favours remodelling, angiogenesis, and regeneration, whereas excessive M1 activity can lead to degeneration, fibrosis, and impaired repair.

Macrophages play a pivotal role in regulating immune responses and tissue remodelling, making them a key target in the design of immunomodulatory biomaterials. Their remarkable phenotypic plasticity allows them to adapt their functional roles dynamically in response to changes in the local microenvironment [20,21]. Depending on environmental cues, macrophages (M0) can polarize into the pro-inflammatory M1 phenotype, which combats infection and initiates inflammation, or the anti-inflammatory M2 phenotype, which promotes tissue repair and regeneration [22] (Figure 3A). M1 macrophages, also known as classically activated macrophages, are distinguished by elevated expression of surface markers including major histocompatibility complex (MHC) class II, CD80, CD86, CD38, CD40 and Toll-like receptor 4 (TLR4). MHC class II is highly expressed on M1 macrophages and plays a central role in antigen presentation to CD4^+^ T cells, thereby promoting adaptive immune activation and sustaining inflammatory responses [23]. The CD80 and CD86 surface markers interact with CD28 and CTLA-4 receptors on T cells, providing essential co-stimulatory and co-inhibitory signals. Engagement with CD28 promotes T-cell activation, proliferation, and cytokine secretion, whereas interaction with CTLA-4 suppresses T-cell responses, thereby maintaining immune balance and limiting excessive inflammation [24]. CD38, a multifunctional ectoenzyme, contributes to inflammatory metabolic reprogramming in M1 macrophages by regulating NAD^+^ metabolism and calcium signalling, thereby supporting key effector functions such as pro-inflammatory cytokine production, reactive oxygen species (ROS) generation, and enhanced antimicrobial activity [25,26]. CD40 is a co-stimulatory receptor highly expressed on M1 macrophages that interacts with CD40L on activated T cells to enhance antigen presentation and amplify pro-inflammatory signalling. CD40 activation promotes the production of cytokines such as TNF-α and IL-12 and strengthens macrophage-mediated immune activation, thereby linking innate and adaptive immune responses [27]. TLR4 is a pattern recognition receptor that detects pathogen-associated molecular patterns, such as lipopolysaccharide (LPS), and activates downstream signalling pathways including NF-κB and MAPK, leading to the production of pro-inflammatory cytokines such as TNF-α, IL-1β, and IL-6 [28]. These cytokines, together with chemokine receptors such as CCR7, contribute to enhanced antigen presentation, increased co-stimulatory signalling, and robust immune activation, which are hallmark features of M1 macrophages [29,30,31].

In contrast, M2 macrophages counterbalance the actions of M1 macrophages by promoting tissue repair, immune regulation, and parasite clearance. The cytokine profile shifts from pro-inflammatory cytokines to anti-inflammatory cytokines, characterized by decreased secretion of IL-12 and IL-23 and increased release of IL-10 and IL-1RA. M2 macrophages also contribute to tissue remodelling, angiogenesis, and modulation of allergic responses [32]. This phenotype is further defined by the production of transforming growth factor-β (TGF-β), vascular endothelial growth factor (VEGF), and epidermal growth factor (EGF), together with elevated expression of surface markers such as CD206, CD163, and CD204, and increased intracellular expression of arginase-1 (ARG1) [33,34,35]. ARG1 metabolizes L-arginine into ornithine and urea, supporting the synthesis of polyamines and proline, which are essential for extracellular matrix formation, collagen deposition, and tissue repair. By depleting L-arginine, ARG1 also limits substrate availability for pro-inflammatory pathways such as nitric oxide production, thereby promoting the resolution of inflammation and creating a regenerative microenvironment [36,37]. CD206, also referred to as the mannose receptor, is a transmembrane protein predominantly expressed on macrophages and dendritic cells. It facilitates the phagocytic and endocytic uptake of bacterial, protozoal, fungal, and viral antigens, thereby contributing to the regulation of innate immunity, modulation of inflammatory responses, and maintenance of tissue homeostasis [38,39]. CD163 is a transmembrane scavenger receptor expressed on monocytes and macrophages that plays a key role in resolving inflammation. It aids in the removal of damaged cells and promotes anti-inflammatory signalling by stimulating the release of anti-inflammatory cytokines and facilitating hemoglobin uptake, which further triggers anti-inflammatory pathways in macrophages [40,41]. CD204 is a scavenger receptor that contributes to multiple physiological and pathological processes, including the modulation of inflammatory responses, innate immune function, host defence, and tumour progression, through its interactions with specific ligands in vivo [42,43].

While initial inflammation is crucial for healing [44], chronic inflammation can lead to persistent tissue damage and impaired tissue regeneration [45]. An ideal immune response involves an early, transient pro-inflammatory phase to clear debris and pathogens, followed by a timely transition to an anti-inflammatory phase that promotes tissue remodelling, angiogenesis, and regeneration (Figure 3B). Dysfunctional immune responses can disrupt the healing process, leading to excessive production of pro-inflammatory signals and growth factors, as well as impaired communication between leukocytes, endothelial cells, and stem cells. However, local cues such as the clearance of apoptotic cells and anti-inflammatory cytokines can promote the polarization of macrophages towards an M2 phenotype, facilitating the resolution of inflammation and tissue repair [46,47]. Experimental studies of bone regeneration show that an early M1-to-M2 shift supports effective healing [48]. Dysregulation of macrophage polarization contributes to pathological conditions, including chronic wounds, autoimmune disorders, and implant-associated inflammation, where excessive M1 activity impedes healing [49]. For example, in skin wound healing, prolonged pro-inflammatory dominance sustains TNF-α, IL-1β, and IL-6 production, delaying closure and promoting fibrotic tissue formation [50,51]. In contrast, the anti-inflammatory phase is marked by IL-10 and TGF-β production, which supports inflammation resolution, angiogenesis, and extracellular matrix remodelling, with IL-10 playing a key role in limiting immune overactivation and maintaining tissue homeostasis [52].

These examples highlight that successful tissue healing depends not only on eliminating pathogens or debris but also on precisely regulating the timing and magnitude of inflammatory and anti-inflammatory phases. Because implanted or engineered materials inevitably interact with the host immune system, their design must take into account how they influence macrophage behaviour and inflammatory signalling.

To support this tightly regulated immune-mediated healing process, biomaterials should be designed to modulate immune responses, limit infection, and promote tissue healing. For example, coating dental implant surfaces with bioactive materials can provide the necessary osteogenic factors to facilitate integration with bone [53]. Recent advances in cell membrane-encapsulated biomaterials for tissue repair illustrate that these engineered systems can mimic the source cells’ surface proteins and functional moieties, enabling improved biocompatibility, reduced immune clearance, and enhanced regenerative capacity [54].

Macrophages play a central role in regulating inflammation and tissue repair through their dynamic polarization. Accordingly, strategies that harness macrophage-derived signals have gained increasing attention in biomaterial design. An emerging approach is the use of macrophage cell membranes to functionalize NPs, allowing synthetic carriers to inherit key immunological properties of macrophages, such as immune recognition, cytokine interactions, and inflammation targeting.

Although the M1/M2 framework remains a useful conceptual tool, macrophage polarization in vivo exhibits far greater plasticity and context-dependency than this rigid binary model suggests. Recent single-cell transcriptomic profiling has uncovered distinct reparative macrophage subpopulations, including CD301b+ and ICAM1+ subsets, which do not conform to classical M1/M2 definitions but actively orchestrate bone regeneration and periodontal tissue repair [55]. Acknowledging this complexity is clinically vital; therapeutic interventions aimed exclusively at canonical M2 polarization markers risk overlooking the broader spectrum of regenerative macrophage phenotypes. Future MMNP formulations should therefore exploit the unique membrane proteomes of these specialized subsets to achieve targeted osteoimmunomodulation, particularly in compromised patient populations, such as individuals with diabetes or immunodeficiency, who are characterized by dysregulated macrophage function.

## 3. Immunomodulatory Applications of Macrophage Membrane-Coated Nanocarriers

The initial development of NPs coated with leukocyte membranes was pioneered by Parodi et al., who fabricated nanoporous silicon NPs cloaked in leukocyte membranes for targeted drug delivery. This early work demonstrated how leukocyte membranes could endow NPs with natural adhesion and signalling properties to overcome vascular barriers, a concept later extended specifically to macrophage membranes for targeted immunomodulatory applications [56]. Since their inception, leukocyte membrane-coated nanoparticles have evolved from primarily bio-interfacing and drug delivery platforms to applications involving immunomodulation and immunosuppression [57,58]. Among leukocyte-derived coatings, MMNPs have gained prominence due to macrophages’ intrinsic roles in immune regulation, tissue remodelling, pathogen clearance, and homeostasis.

Natural cell membranes preserve a broad range of functional proteins and receptors from their parent cells, thereby maintaining their biological identity and functionality [59]. Membrane-camouflaged nanoparticles combine the structural advantages of synthetic NPs with the biological functionality of natural cell membranes, thereby mimicking the interactions of their parent macrophages with the microenvironment [60].

When synthetic NPs are cloaked in macrophage membranes, they acquire immune-evasive properties that enable them to avoid immune recognition and reduce phagocytic clearance. Beyond immune escape, this biomimetic coating preserves key macrophage-derived surface proteins and receptors, allowing the NPs to interact dynamically with inflammatory mediators and local immune microenvironments. As a result, MMNPs can replicate several intrinsic immune functions of macrophages, including the sequestration of pro-inflammatory cytokines, neutralization of endotoxins, and modulation of dysregulated inflammatory signalling pathways.

The functional behaviour of MMNPs is strongly influenced by macrophage polarization states, which determine their immunological phenotypes and downstream biological activities. Accordingly, MMNPs can be engineered using membranes derived from different macrophage phenotypes (M0, M1, or M2), allowing tailored therapeutic functions depending on the disease context. Within this framework, M2 macrophage membrane-coated nanoparticles (M2-MMNPs) exhibit prominent anti-inflammatory and tissue-regenerative properties. By retaining M2-associated surface proteins and receptors, M2-MMNPs promote immune suppression, resolution of inflammation, and tissue repair [61,62,63]. For example, Nakkala et al. investigated macrophage membrane-coated nanofibers derived from different polarization states (M0, M1, and M2). Among these, M2-coated nanofibers (M2-PCL) demonstrated superior immunomodulatory effects, including the promotion of macrophage polarization toward the M2 phenotype, downregulation of pro-inflammatory chemokines, and enhancement of anti-inflammatory responses in both in vitro and in vivo models, highlighting the importance of phenotype selection for regenerative applications [62].

Beyond inflammation control, MMNPs have also gained increasing attention in tissue regeneration and repair applications, where the precise regulation of macrophage-mediated inflammation is essential for successful healing. In this context, the macrophage-derived membrane enables these nanocarriers to function as biological decoys, interacting with and modulating components of the inflammatory microenvironment, including damage-associated molecular patterns (DAMPs), thereby supporting the resolution phase of tissue repair. Owing to the intrinsic plasticity of macrophages, MMNPs can be further engineered to achieve context-dependent immunomodulation, enabling either pro-inflammatory activation or anti-inflammatory responses, depending on the therapeutic requirements.

One critical vulnerability warranting deeper interrogation is the phenotypic fidelity of the membrane during fabrication. Although the prior literature highlights clear advantages of M2-derived membranes in regenerative medicine, the degree to which standard coating methodologies—namely sonication, extrusion, or electroporation—compromise or preserve the proteomic integrity and functional receptor landscape of the source macrophage remains under-characterized. This unquantified batch-to-batch variability introduces significant reproducibility challenges across the published literature. We propose that quantitative proteomic benchmarking should become a standardized quality control strategy to verify that MMNPs preserve their functional membrane proteome beyond the lipid bilayer architecture. Without these metrics, cross-study comparisons are obfuscated and the therapeutic efficacy of individual MMNP formulations cannot be rigorously validated. Therefore, fully realizing the therapeutic potential of MMNPs requires a holistic approach that optimizes both this outer biological shell and the inner synthetic engine. While the membrane coating imparts vital targeting, immunomodulatory, and protective properties, the performance of these nanocarriers also critically depends on the underlying nanoparticle core including its composition, size, surface characteristics, and responsiveness to environmental stimuli. Understanding and optimizing these core material properties is essential to complement surface bio-functionality, which will be discussed in detail in the following section.

## 4. Core Material Properties of Macrophage Membrane-Coated Nanoparticles

The properties of core materials employed in macrophage membrane-coating strategies are critical determinants of coating efficiency and the resulting immunomodulatory performance. These NPs can be fabricated from both natural biomaterials, such as chitosan, alginate, and silk fibroin, as well as from synthetic systems, including polymeric, lipid-based, and metallic structures. The selection of core materials depends largely on the intended biological function, stability, and compatibility with the macrophage membrane. In recent years, a wide range of core materials, including poly lactic-co-glycolic acid (PLGA) [64,65], SiO_2_ [66], iron oxide [67], nanogels [68], metal–organic frameworks (MOFs) [69], mesoporous silica nanocapsules (MSNs) [70] and liposomes [71], have been explored for this purpose.

Prior to in vivo application, a comprehensive evaluation of these materials is essential. The physicochemical properties of core NPs including size, shape, surface charge, elasticity, curvature, and chemical composition play a critical role in determining both membrane coating efficiency and subsequent biological interactions. Uniform and well-defined nanoparticle structures are generally preferred, as they facilitate consistent membrane coverage and stable encapsulation. These structural and surface characteristics influence key parameters such as surface area, size-to-volume ratio, and mechanical strength, which in turn affect cellular uptake, bio–nano interactions, and the long-term stability of the membrane coating. Therefore, careful optimization of these properties is necessary to achieve reliable coating quality and predictable biological performance [72].

Among these physicochemical characteristics, nanoparticle morphology plays a particularly important role in membrane–nanoparticle interactions because successful membrane coating requires deformation and wrapping of the lipid bilayer around the nanoparticle core. Spherical NPs are generally considered the most favorable geometry because their uniform curvature facilitates homogeneous membrane wrapping and minimizes membrane stress during extrusion or sonication [73]. In contrast, rod-shaped NPs possess regions of non-uniform curvature that require greater membrane deformation and higher adhesion energy for complete membrane wrapping than spherical NPs, potentially reducing wrapping efficiency. These findings suggest that nanoparticle geometry is an important design parameter governing membrane–nanoparticle interactions and may influence the efficiency and uniformity of cell membrane coating [74].

The size and composition of NPs are key factors influencing the stability, integrity, and performance of cell membrane-coated nanosystems. NPs with diameters ranging from 65 to 340 nm have been successfully coated, and tailoring NP size can optimize properties such as cargo loading, circulation time, and tissue penetration [75,76]. Nanoparticle size also affects membrane coating efficiency. Smaller particles require less bending energy for membrane wrapping, enabling more complete and uniform coating compared to larger particles [77,78,79]. Moreover, size influences cellular uptake, biodistribution, and in vivo circulation. For example, erythrocyte membrane-coated nanoparticles of 80 nm circulated longer and crossed biological barriers more efficiently than larger particles, which tended to accumulate in the liver and spleen [14]. Although MMNPs are increasingly developed for biomedical applications, studies systematically assessing the influence of nanoparticle size on coating efficiency and uniformity remain limited. Nevertheless, findings from other cell membrane-coated systems indicate that optimizing nanoparticle size is likely critical for achieving uniform coating, prolonged circulation, and enhanced therapeutic performance in macrophage-based biomimetic nanocarriers.

The surface charge of NPs plays a critical role in the successful synthesis of membrane-coated NPs. Due to the asymmetric charge distribution of cell membranes, electrostatic interactions between the membrane and nanoparticle surface strongly influence coating efficiency and membrane orientation during fusion. Negatively charged NPs generally support more stable membrane coatings, whereas positively charged NPs are prone to aggregation. Cell membranes are rich in negatively charged sialyl moieties on their outer surface. As a result, positively charged NPs exhibit strong electrostatic attraction to the membrane, which can lead to disruption of the lipid bilayer and collapse of the membrane structure, preventing proper coating. In contrast, negatively charged NPs experience electrostatic repulsion with the membrane’s outer surface, minimizing membrane distortion and promoting a more uniform and stable coating. Moreover, a negative surface charge not only reduces non-specific interactions but also facilitates the correct orientation of the membrane during the fusion process, further enhancing coating efficiency and structural integrity [80].

These physicochemical interactions can be experimentally verified using standard characterization techniques. Successful wrapping can be validated by zeta potential measurements, as the potential of MMNPs should approach that of the native macrophage membrane [81,82]. This shift in surface charge confirms successful membrane transfer onto the nanoparticle core. In addition, membrane coating is commonly accompanied by a measurable increase in hydrodynamic diameter. For example, Wei et al. reported a 10–15 nm increase in particle size after membrane coating compared to uncoated NPs, further confirming successful membrane encapsulation [83].

Beyond surface charge considerations and validation, optimization of fabrication parameters is also essential to achieve consistent coating quality. In particular, the membrane-to-core ratio is a critical determinant of coating efficiency and stability. Wang et al. [84] demonstrated that coating zeolitic imidazolate framework-8 NPs with macrophage-derived microvesicles at an optimized ratio of 2:1 resulted in NPs with a well-defined core–shell structure, uniform size, and stable surface charge following sonication and membrane extrusion.

Nanoparticle concentration (density) is a key parameter influencing the efficiency and uniformity of cell membrane coating. Effective membrane wrapping relies on sufficient collisions between membrane vesicles and nanoparticle cores. For example, in one study, Yang et al. optimized the coating of PLGA nanoparticles with plasma membrane vesicles using sonication and found that low nanoparticle densities (e.g., 0.5 mg/mL) resulted in incomplete coating, larger particle sizes, and higher polydispersity, indicating poor coating efficiency and colloidal instability. In contrast, densities in the range of 1–3 mg/mL and sample volumes between 100 and 400 µL produced uniform and stable membrane-coated nanoparticles, as more frequent vesicle–core collisions occurred under these conditions [85]. Although similar systematic studies have not yet been reported for MMNPs, these findings highlight a general principle: maintaining adequate nanoparticle concentration is critical for reproducible and efficient membrane coating across biomimetic systems.

The mechanical properties of nanoparticle cores, notably their elasticity or stiffness, are emerging as important determinants of biomimetic nanocarrier behaviour. Studies have shown that softer NPs may favour more complete or protein-rich membrane coatings and reduced uptake by macrophages, whereas stiffer particles may behave differently in terms of cellular uptake, blood circulation, and tumour targeting efficiency [86,87,88]. To elucidate the role of NP elasticity in the context of CMCNPs, Zou et al. investigated mesenchymal stem cell (MSC) membrane-coated silica nanoparticles (MCSNs) with similar size but significantly different stiffness [89]. Softer MCSNs promoted the formation of a more extensive, protein-rich membrane coating, characterized by higher levels of the MSC-specific chemokine CXCR4 and surface marker CD90, indicating that NP elasticity can directly affect coating quality and cellular interactions. While direct measurements of stiffness effects in MMNPs are still limited, these findings from other cell membrane systems suggest that tuning nanoparticle stiffness may be a viable strategy for optimizing immunomodulative biomimetic carriers.

**Figure 4:**
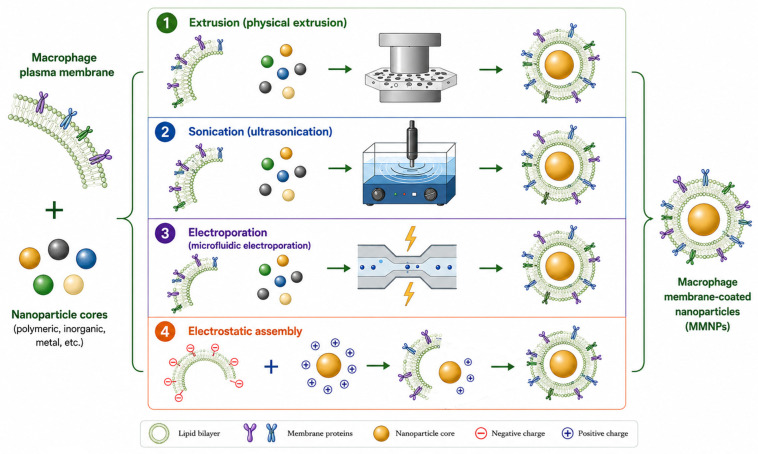
Schematic illustration of the major fabrication strategies used to prepare macrophage MMNPs, including physical extrusion, sonication, electroporation, and electrostatic assembly. Macrophage plasma membranes are combined with nanoparticle cores to generate biomimetic NPs that retain membrane-associated proteins and biological functions.

In addition to core composition, the fabrication process strongly influences the structure and biological performance of MMNPs. Efficient coating of nanoparticle cores with macrophage-derived membranes is required in order to preserve functional surface proteins and receptors [90]. Several fabrication methods have been developed, including physical extrusion, sonication, electroporation, and electrostatic assembly (Figure 4).

Physical extrusion involves passing NPs and membrane vesicles through porous membranes to promote membrane fusion and generate uniformly coated NPs [63,77]. Despite its advantages, scaling up this method remains challenging due to material losses caused by filter deposition, which makes the process labour-intensive and less suitable for industrial-scale applications [91]. Sonication uses ultrasonic energy to facilitate membrane reassembly around nanoparticle cores and is valued for its simplicity and rapid processing; however, excessive ultrasonic force may compromise membrane integrity and coating homogeneity [85]. Electroporation employs pulsed electric fields to transiently permeabilize macrophage membranes, enabling nanoparticle encapsulation with improved control over particle size and coating efficiency. Nevertheless, excessive electrical stimulation may disrupt membrane proteins and alter membrane functionality [92,93]. Electrostatic assembly relies on charge interactions between NPs and membrane components to promote coating formation. Comparative studies indicate that physical extrusion generally provides the most complete and uniform membrane coverage while better preserving the biological activity of macrophage membrane proteins than other fabrication methods [94,95], and it remains the preferred and most reliable approach for producing high-quality MMNPs due to its reproducibility, scalability, and ability to maintain membrane structural and functional integrity [96].

Despite the substantial advances in core material engineering described above, a critical gap persists: the lack of standardized fabrication protocols. The majority of the published MMNP literature relies on institution-specific methodologies with highly variable membrane-to-core ratios, sonication parameters, and extrusion parameters, rendering cross-study comparisons challenging. This methodological heterogeneity is not merely a logistical challenge; it represents a fundamental bottleneck to clinical translation, given that regulatory agencies mandate highly reproducible, well-defined manufacturing processes for cell-derived therapeutics. Consequently, the field should establish community-agreed standard operating procedures (SOPs) for membrane isolation, nanoparticle assembly, and quality control. Implementing minimum reporting standards analogous to the Minimal Information for Studies of Extracellular Vesicles (MISEV) guidelines established by the International Society for Extracellular Vesicles (ISEV) is an essential prerequisite to unlocking the translational potential of these biomimetic formulations.

## 5. Therapeutic Applications of Macrophage Membrane-Coated Nanoparticles

Macrophage membrane-coated nanoparticles have emerged as a versatile platform for targeted therapy due to their ability to evade immune clearance, modulate inflammation, and enhance tissue-specific delivery. In this section, we discuss their therapeutic applications in several areas, including inflammatory disorders, tissue regeneration, and oral health, with a focus on dental implant-related applications.

### 5.1. Inflammatory Disorders

Inflammatory disorders are characterized by dysregulated immune responses, excessive pro-inflammatory cytokine production, leukocyte overactivation, and subsequent tissue damage. Conventional therapies are often limited by systemic toxicity, off-target effects, and insufficient localization to disease sites [97].

In this context, MMNPs represent a promising biomimetic strategy by combining the intrinsic targeting ability of macrophages with the functional versatility of nanomaterials. By mimicking macrophage surface proteins and receptors, MMNPs can selectively accumulate at inflamed sites, bind to overexpressed adhesion molecules such as ICAM-1 and VCAM-1, neutralize pro-inflammatory mediators, and modulate immune responses, thereby reducing tissue injury and enhancing therapeutic efficacy. For example, macrophage biomimetic PLGA nanoparticles (M0-NPs) have been designed to mimic macrophage functions and combat systemic inflammation by neutralizing toxins, sequestering pro-inflammatory cytokines, and preventing tissue damage. In animal models, M0-NP treatment reduced systemic cytokine levels, limited inflammatory spread, and significantly improved survival [19].

Inflammation-associated bone loss and impaired tissue healing are major challenges in oral and maxillofacial applications, including peri-implantitis and implant failure. MMNPs may therefore offer significant therapeutic advantages by reshaping the inflammatory microenvironment, reducing cytokine-mediated tissue destruction, and promoting tissue regeneration. Among macrophage-based biomimetic systems, MMNPs derived from M2 macrophages have demonstrated particular potential to induce anti-inflammatory responses and support tissue repair through immunomodulatory mechanisms. For instance, macrophage-biomimetic porous selenium–silica nanocomposites (M-Se@SiO_2_) have been shown to suppress LPS-induced inflammation, promote M2 macrophage polarization, and restore osteogenic activity through the inhibition of p65, p38, and ERK signalling pathways, thereby enhancing bone regeneration in vitro and in vivo [98].

In particular, M2 macrophage membrane-based systems have been reported to regulate macrophage polarization, suppress pro-inflammatory cytokine expression, and support tissue remodeling processes relevant to bone healing and regenerative applications. Key studies in this area are summarized in Table 1. These systems have demonstrated broad immunomodulatory and regenerative potential across different biomaterial platforms, including nanofibers and scaffold-based constructs, highlighting their relevance for tissue repair strategies.

Collectively, these findings underscore the critical role of macrophage phenotype engineering in regulating osteoimmunomodulatory responses. MMNPs have emerged as a highly promising class of biomimetic platforms capable of controlling inflammation and enhancing tissue regeneration, particularly for bone repair and implant-associated inflammatory conditions in dental settings. While the reviewed in vivo studies consistently report that MMNPs reduce pro-inflammatory cytokine profiles and improve tissue outcomes, a critical appraisal of the literature reveals a significant mechanistic gap. Most published investigations primarily rely on broad cytokine panels as primary readouts and fail to determine whether the observed therapeutic benefits arise predominantly from cytokine sequestration, active macrophage repolarization, or direct anti-endotoxin activity. This distinction is clinically important because different pathological environments such as sterile peri-implant osteolysis and bacterially driven peri-implantitis are likely to require distinct mechanisms of MMNP action. Future studies should therefore incorporate pathway-specific functional assays (e.g., NF-κB reporter systems and phosphoproteomic analyses) to better define these underlying mechanisms. Furthermore, the current reliance on simplified LPS-induced models may overestimate MMNP efficacy; therefore, future MMNP formulations should be evaluated in more complex polymicrobial environments that more accurately reflect clinical conditions.

### 5.2. Tissue Regeneration

Tissue regeneration relies heavily on the dynamic interplay among immune regulation, angiogenesis, and extracellular matrix remodelling, in which macrophages play a central role. Controlled modulation of macrophage polarization from the pro-inflammatory M1 phenotype to the pro-healing M2 phenotype is critical for orchestrating effective tissue repair. MMNPs have emerged as a promising strategy to promote this immunoregulatory balance while delivering bioactive molecules that accelerate regeneration.

Bone regeneration is tightly regulated by the immune system, particularly by macrophages, which coordinate inflammation, angiogenesis, and extracellular matrix remodeling. An early inflammatory response is necessary to initiate healing, but excessive or prolonged inflammation impairs osteogenesis and delays effective tissue repair. Therefore, strategies that modulate immune responses while supporting osteogenic activity are critical for improving bone healing. These immunoregulatory approaches are especially relevant for clinical applications such as dental implants, where successful osseointegration depends on balanced inflammation and efficient bone regeneration.

In this context, MMNPs have been explored as multifunctional platforms for immunomodulation and bone regeneration. For example, M-Se@SiO_2_ nanoparticles have been developed to treat inflammatory osteolysis. These nanocomposites act as macrophage membrane decoys, enabling the neutralization of endotoxins such as LPS and the sequestration of pro-inflammatory cytokines. In parallel, the release of selenium promotes macrophage polarization toward the anti-inflammatory M2 phenotype, thereby contributing to the formation of a pro-regenerative microenvironment.

As a result, M-Se@SiO_2_ nanospheres attenuate inflammation and alleviate the inhibitory effects of pro-inflammatory cytokines on osteogenic differentiation, as demonstrated in both in vitro and in vivo models. These findings highlight the potential of MMNP-based systems to simultaneously regulate the immune microenvironment and support bone regeneration, aligning with the key requirements for the treatment of inflammatory bone loss [98].

In bone tissue engineering, biomimetic anti-inflammatory nanocapsules (BANCs) coated with macrophage membranes and loaded with resolvin D1 (RvD1) were developed to modulate the local immune environment. Activated by near-infrared (NIR) light, these NPs sequestered pro-inflammatory cytokines and induced M2 polarization, ultimately promoting bone regeneration in mouse models [102]. Hybrid matrices that combine M2 macrophage membranes with osteoinductive mesenchymal stem cell (oiMSC) membranes further illustrate the therapeutic versatility of MMNP-inspired biomaterials. Qiao and Lv developed a hybrid cell membrane-functionalized matrix (MFM) by coating poly(ε-caprolactone) (PCL) electrospun nanofibers with membranes derived from M2-polarized macrophages and osteogenically induced MSCs (1:1). The resulting MFMs suppressed pro-inflammatory cytokines (TNF-α, IL-6, and IL-1β), promoted anti-inflammatory cytokines (IL-10, TGF-β1, and IL-1ra), enhanced M2 macrophage polarization, and upregulated osteogenic markers (ALP, OPN, OC, RUNX2, and COL I), leading to improved mineralization and accelerated bone regeneration both in vitro and in a rat calvarial defect model. These findings demonstrate the therapeutic potential of combining immunomodulatory and osteogenic membrane functions within a single biomimetic platform for coordinated tissue regeneration [99].

Concurrently, these studies demonstrate that macrophage membrane-coated nanoplatforms can effectively regulate inflammatory responses while simultaneously promoting osteogenesis. By modulating macrophage polarization and accelerating bone formation, these biomimetic systems establish a highly favourable microenvironment for osseointegration. Such immunomodulatory strategies hold immense therapeutic potential for dental implant applications, in which suppressing destructive inflammation and ensuring robust bone–implant integration are paramount to preventing clinical failure.

However, despite this encouraging preclinical evidence, major translational bottlenecks remain unaddressed. First, the current reliance on healthy rodent models with standardized defect geometries fails to recapitulate the complex microbial burden, chronic inflammation, and compromised healing capacity seen in clinical patients, particularly those with diabetes, osteoporosis, or a history of implant failure. Second, standard short-term endpoints (e.g., ALP activity, RUNX2 expression, and 4–8-week histomorphometry) reflect early osteogenic activity but offer negligible insight into long-term implant stability or mature bone quality. Third, the dual-functional nature of MMNPs which concurrently drives immunomodulation and delivers osteogenic cargo frequently confounds mechanistic interpretation, making it difficult to isolate the therapeutic contributions of each component. To responsibly advance these platforms toward clinical translation, the field must adopt highly representative pathological models (including diabetic and senescent animals), extend evaluation timelines, and implement factorial experimental designs that rigorously decouple membrane-mediated immunity from cargo-driven bone regeneration.

### 5.3. Oral Diseases and Dental Implant Applications

Oral diseases such as periodontitis and peri-implantitis are primarily initiated by bacterial dysbiosis and biofilm formation at the tooth or implant surface. Pathogenic bacteria, particularly *Porphyromonas gingivalis* (*P. gingivalis*), trigger a persistent inflammatory response by releasing virulence factors such as lipopolysaccharides, which activate immune cells and amplify local cytokine production [103]. This sustained inflammatory environment disrupts tissue homeostasis and leads to the progressive destruction of periodontal tissues and alveolar bone. In the context of dental implants, excessive inflammation can impair osseointegration by inhibiting osteogenic activity and promoting bone resorption around the implant surface. Therefore, controlling both bacterial infection and the associated inflammatory response is essential for maintaining tissue integrity and ensuring successful implant integration.

**Figure 5:**
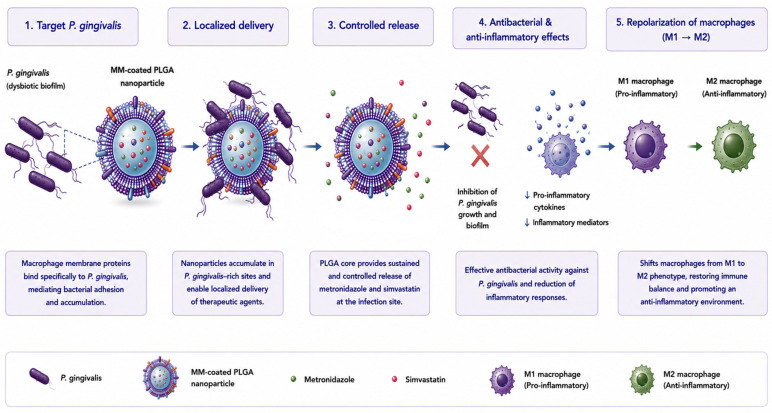
Schematic illustration of the therapeutic mechanism of macrophage membrane-coated poly (lactic-co-glycolic acid) (PLGA) NPs co-loaded with metronidazole and simvastatin for the treatment of *Porphyromonas gingivalis* (*P. gingivalis*)-associated infection. Macrophage membrane coating enables specific targeting and adhesion to *P. gingivalis*-rich sites, facilitating localized delivery of therapeutic agents. The PLGA core provides a sustained and controlled release of metronidazole and simvastatin, resulting in effective antibacterial and anti-inflammatory activity. This strategy inhibits *P. gingivalis* growth and biofilm formation while promoting macrophage repolarization from the pro-inflammatory M1 phenotype to the anti-inflammatory M2 phenotype, thereby restoring immune homeostasis and suppressing inflammation.

Recent advances have highlighted the potential of MMNPs in immunomodulation for oral diseases and dental implant applications. These biomimetic systems exploit the immune-regulatory functions of macrophage membranes to target inflammatory microenvironments, control infection, and promote tissue healing. For instance, macrophage membrane-coated poly (lactic-co-glycolic acid) (PLGA) nanoparticles co-loaded with metronidazole and simvastatin were engineered to target *P. gingivalis*. The macrophage membrane facilitated specific bacterial adhesion and localized drug delivery, enabling controlled release and effective inhibition of disease progression. This nanosystem exhibited sustained drug release, significant antibacterial and anti-inflammatory effects in vitro, and efficiently attenuated inflammation in a Pg-induced mouse model by repolarizing M1 macrophages toward the M2 phenotype [104] (Figure 5). In another study, macrophage membrane-coated silk fibroin nanoparticles (MSNCs) overexpressing TLR4 were designed to recognize and neutralize bacterial LPS. These MSNCs acted as dual-function nanoplatforms, targeting and killing pathogenic bacteria while serving as decoys to reduce LPS-mediated immune activation. In vitro results demonstrated selective bacterial targeting and suppression of inflammatory macrophage activation. Furthermore, in vivo experiments in an LPS-induced periodontitis mouse model showed that MSNC treatment markedly reduced periodontal inflammation and alveolar bone loss, highlighting their potential as a synergistic therapy for periodontitis beyond conventional treatments [105].

Building on this concept, macrophage membrane-coated biomaterials have also been developed to regulate the inflammatory microenvironment associated with periodontal disease and bone loss. A recent study developed macrophage membrane-coated poly (lactic-co-glycolic acid)/β-tricalcium phosphate (PLGA/β-TCP) composite microspheres modified with poly-L-lysine (PLL), termed MM@PPT microspheres, to promote alveolar bone regeneration in periodontitis. In this design, macrophage membranes activated by lipopolysaccharide and interferon-γ (IFN-γ) were used to endow the microspheres with the ability to neutralize proinflammatory factors and modulate immune cell behavior. The MM@PPT microspheres effectively inhibited macrophage M1 polarization and osteoclast differentiation while promoting M2 polarization and osteogenic differentiation of bone marrow stromal cells (BMSCs), even under inflammatory conditions. When injected into sites of bone resorption in a periodontitis model, the microspheres significantly enhanced alveolar bone regeneration by restoring the immune–regeneration balance. These findings underscore the therapeutic potential of macrophage membrane-coated microspheres for immunomodulatory regeneration in periodontal disease, offering a promising strategy for improving bone healing and implant integration in oral applications [106].

While macrophage membrane-coated nanoparticles represent a promising biomimetic strategy to modulate the immune microenvironment and enhance tissue regeneration in periodontitis, their clinical validation remains limited by a lack of representative oral infection models. Peri-implantitis is a complex, polymicrobial, biofilm-driven disease operating under constant mechanical loading, dynamic features that are completely absent from static, single-pathogen LPS models. Furthermore, nanoparticle retention within the highly specialized peri-implant niche (encompassing bone, epithelial, and connective tissue boundaries) presents unique spatial challenges. Consequently, the most viable translational pathway for MMNPs in dentistry is local, site-specific application as an adjunct during implant installation or surgical debridement. Delivering targeted immunomodulation directly to the compromised tissue interface offers a practical method to complement existing mechanical therapies, and this localized clinical integration should guide future study designs.

### 5.4. Emerging Macrophage-Based Nanoplatforms for Precision Immune Modulation in Dental and Implant-Related Inflammation

Beyond conventional macrophage membrane-coated nanoparticles, recent advances have introduced next-generation macrophage-inspired nanoplatforms, which further expand the scope of immune modulation and therapeutic precision. These systems include exosome-based platforms, stimuli-responsive nanoparticles, and gene-editing nanocarriers that enable dynamic and context-dependent regulation of inflammatory microenvironments.

For example, macrophage-derived exosomes and exosome–nanoparticle hybrids retain intrinsic homing capabilities and can deliver regulatory microRNAs or small interfering RNAs to modulate inflammatory signalling pathways at the transcriptional level [107]. In parallel, reactive oxygen species (ROS)-responsive nanoparticles and nanozyme-based platforms have been engineered to regulate oxidative inflammatory microenvironments through controlled drug release and enzymatic ROS scavenging, thereby enhancing therapeutic efficacy while reducing systemic toxicity [108,109]. These oxidative stress-associated mechanisms are also relevant in chronic oral inflammatory conditions, including periodontal and peri-implant diseases [110,111], highlighting the potential applicability of these ROS-responsive nanoplatforms for oral inflammatory conditions.

In addition, nucleic acid-based delivery systems, including mRNA and CRISPR/Cas nanocarriers, have been developed to regulate key inflammatory mediators such as NLRP3 and IL-1β, thus enabling precise control of inflammatory signalling pathways [112]. These pathways are also implicated in peri-implant bone resorption and periodontal inflammation, highlighting their relevance to dental disease pathogenesis.

Furthermore, immunometabolic reprogramming strategies have shown promise in shifting macrophage metabolism toward anti-inflammatory phenotypes, thereby promoting resolution of chronic inflammation and supporting tissue regeneration [113]. These mechanisms may also be advantageous in the context of dental and craniofacial tissue repair, in which immune balance is essential for successful healing and implant integration.

The integration of programmable, multifunctional strategies with macrophage membrane-based nanoplatforms marks a definitive shift toward active immunomodulation, offering much-needed spatio-temporal control for managing dental peri-implantitis and bone regeneration. However, this escalating complexity exposes a fundamental conceptual gap: a biomimetic paradox. As we increasingly engineer these membranes with synthetic components, stimuli-responsive links, and gene-editing machinery, we progressively compromise the native cellular identity that grants them immune-evasion. A heavily modified biological shell risks being recognized as foreign, potentially inciting the exact immune clearance it was engineered to avoid. Beyond this biological uncertainty lies a daunting translational hurdle: every added feature shifts the regulatory landscape, causing a single formulation to cross into drug, biological, and gene therapy jurisdictions. Moving these platforms from bench to clinic requires the bio-materials community to move past mere proof-of-concept studies and actively develop standard product characterization frameworks in alignment with regulatory agencies.

The translational potential of macrophage membrane-coated nanoparticles (MMNPs) extends beyond their immunomodulatory properties to addressing several unresolved clinical challenges in implant dentistry. Their multifunctional design enables the simultaneous modulation of inflammatory signalling, regulation of macrophage polarization, and localized therapeutic delivery, making them promising candidates for managing implant-associated inflammation and promoting tissue regeneration [98,114]. Similarly, aseptic implant loosening driven by wear particle-induced osteolysis can be mitigated via MMNP-mediated delivery of anti-inflammatory nucleic acids targeting the Gas6/Axl or miR-155 axes [115]. Specifically, macrophage membrane-coated nanoparticles delivering an antagomir of miR-155-5p have been shown to mitigate titanium particle-induced osteolysis by modulating the periprosthetic microenvironment via Gas6/Axl signalling, reducing inflammatory responses while promoting osteoblastogenesis. This targeted immunomodulatory support is particularly vital for medically compromised cohorts, such as diabetic patients, who exhibit impaired host macrophage polarization and delayed osseointegration but demonstrate accelerated bone-to-implant contact (BIC) when treated with M2-derived biomimetic coatings [116,117,118].

Because these promising therapeutic claims currently rest entirely on preclinical inference, the field’s immediate priority must shift to executing localized, first-in-human safety and pharmacokinetic studies to validate these platforms. Adjunctive peri-implantitis management during surgical debridement offers an ideal clinical model for these initial trials, as localized delivery minimizes systemic exposure while the primary endpoints of pocket depth reduction and alveolar bone level stabilization are well-defined and quantifiable. These initial human studies are crucial not only to move past theoretical inference but also to generate the baseline biodistribution and immunogenicity datasets mandated by regulatory agencies for clinical approval.

Initiating these human trials, however, first requires resolving three systemic limitations regarding fabrication, model selection, and long-term toxicity that currently limit the field’s maturity. First, fabrication heterogeneity remains a fundamental concern; membrane protein topography varies inherently by macrophage source and extraction method, yet most of the literature omits the comprehensive proteomic characterization needed to decouple specific protein signalling from non-specific lipid bilayer effects. Second, although current preclinical models have provided valuable mechanistic insights into macrophage-mediated osteolysis and implant-associated inflammation, their predictive value for clinically heterogeneous patient populations remains limited because they cannot fully recapitulate the complex biological and inflammatory microenvironment encountered in human disease. Finally, chronic bioaccumulation profiles, immunogenicity following repeated dosing, and potential off-target effects of genetic cargoes remain virtually uncharacterized, representing a significant biological risk.

To overcome these limitations and bridge the translational gap, future innovation must pivot away from empirical formulation and toward precision medicine, advanced manufacturing, and regulatory science. High-resolution single-cell transcriptomic analyses have revealed substantial heterogeneity within reparative macrophage populations, suggesting that future MMNPs could be engineered to selectively target specialized macrophage subsets, such as CD301b+ and ICAM1+ macrophages, rather than relying solely on the conventional M1/M2 paradigm [55,119,120]. Additionally, integrating these macrophage-derived membranes with stimuli-responsive nanoparticle cores activated by near-infrared light, reactive oxygen species (ROS), or pH changes could enable precise spatiotemporal control of immunomodulation, thereby synchronizing therapeutic cargo release with the dynamic phases of tissue healing [121,122]. The acceleration of these sophisticated design iterations will ultimately depend on incorporating computational tools, such as machine-learning-guided optimization of membrane composition and nanoparticle geometry, to systematically predict performance and streamline development.

By pairing these digital screening tools with a strategic clinical focus on site-specific, adjunctive dental applications, the biomaterials community can establish a manageable risk profile and clearer regulatory framework. Ultimately, navigating this inflection point successfully will require the next generation of studies to match the compelling nature of the foundational science with an equal measure of methodological rigour and translation-focused design [123].

## 6. Conclusions

MMNPs represent a promising biomimetic platform that integrates immunological functionality with nanomaterial engineering to enable targeted modulation of inflammatory microenvironments. By preserving key membrane-associated proteins involved in immune recognition and inflammation targeting, MMNPs can selectively interact with activated immune sites, neutralize pro-inflammatory mediators, and regulate macrophage-driven responses. These properties make them particularly relevant for chronic inflammatory conditions, tissue regeneration, and oral diseases such as periodontitis and peri-implantitis, where dysregulated immune activity contributes to disease progression and impaired healing. However, clinical translation remains challenged by issues including scalable and reproducible manufacturing, preservation of membrane functionality, and limited long-term data on biosafety, biodistribution, and pharmacokinetics. Continued interdisciplinary efforts to optimize MMNP design and integrate them with advanced therapeutic strategies, such as tissue engineering, gene-based approaches, and stimuli-responsive systems, are expected to enhance their precision and efficacy. Overall, MMNPs offer significant potential as next-generation nanotherapeutics for immune modulation and hold particular promise for improving outcomes in dental and regenerative applications.

## Figures and Tables

**Table 1:** Summary of the immunosuppressive roles of M2 macrophage membrane-coated nanoparticles.

Cell-Type	Type of Nanoparticle	Functions	Categories	Result	Ref.
Combination of M2 macrophage membranes and osteoinductive mesenchymal stem cells	PCL	Polarized macrophages to anti-inflammatory M2 Reduced expression of MHC I and MHC II molecules Promoted anti-inflammatory cytokine production (IL-10, TGF-β1, IL-1ra) Inhibited pro-inflammatory cytokine production (TNF-α, IL-6, IL-1β) and chemokines (CCR7)	Skull bone defect	Anti-inflammatory Osteoinductive properties	[99]
M2 macrophage	Gelatin and chondroitin sulfate nanogel	Targeted inflamed areas Reduced inflammation Adhered to inflamed chondrocytes via macrophage membrane proteins (CD14, CD44, and Mac-1)	Osteoarthrosis	Cartilage repair	[100]
	KAFAK and shRNA-LEPR condensed with polyethylenimine (PEI)	Significant increase in M2 macrophage markers (CD206, F4/80) in RAW264.7 cells, indicating successful repolarization M2 membrane-coated nanoparticles induced the highest M2 marker expression compared with the control group, demonstrating a conversion from the M1 to the M2 phenotype	Osteoarthrosis	Promotion of cartilage repair	[101]
Different types of macrophages (M0, M1, and M2)	Gold (Au)	Au-M2 macrophages showed: The strongest ability to sequester pro-inflammatory cytokines IL-1β and TNF-α; Reduced IL-1β-induced inflammation and matrix degradation in osteoarthritis (OA); Suppression of transcription of pro-inflammatory genes	Osteoarthrosis	Alleviate inflammation and related damage in OA	[63]
	PCL	PCL-M2 macrophages showed: anti-inflammatory effects by suppressing inflammatory markers and stimulating anti-inflammatory markers Polarization of macrophages to M2 phenotype Neutralization of inflammatory chemokines Inhibition of NF-kB and IRF-5, reducing expression of inflammation-associated genes	Tissue repair and remodeling in vitro and in vivo (subcutaneous tissue on the dorsal (back) surface of mice)	Tissue regeneration and inflammation reduction	[62]

## Data Availability

No new data were created or analyzed in this study.

## Abbreviations

The following abbreviations are used in this manuscript:

ALP	Alkaline phosphatase
ARG1	Arginase-1
Axl	Axl receptor tyrosine kinase
BANC	Biomimetic anti-inflammatory nanocapsule
BMSCs	Bone marrow stromal cells
CCR2	C-C chemokine receptor 2
CCR7	C-C chemokine receptor 7
CD	Cluster of differentiation
CD40L	CD40 ligand
CMCNPs	Cell membrane-coated nanoparticles
COL I	Type I collagen
CRISPR/Cas	Clustered regularly interspaced short palindromic repeats/CRISPR-associated system
CTLA-4	Cytotoxic T-lymphocyte-associated protein 4
CXCR4	C-X-C motif chemokine receptor type 4
DAMPs	Damage-associated molecular patterns
EGF	Epidermal growth factor
ERK	Extracellular signal-regulated kinase
EVs	Extracellular vesicles
Gas6	Growth arrest-specific protein 6
ICAM-1	Intercellular adhesion molecule-1
IFN-γ	Interferon-γ
IL	Interleukin
IL-1RA	Interleukin-1 receptor antagonist
LPS	Lipopolysaccharide
MAPK	Mitogen-activated protein kinase
MCSNs	membrane-coated silica nanoparticles
MHC	Major histocompatibility complex
MiR-155	MicroRNA-155
MMNPs	Macrophage membrane-coated nanoparticles
M2-MMNPs	M2 macrophage membrane-coated nanoparticles
MOFs	Metal–organic frameworks
MSC	Mesenchymal stem cell
MSNCs	Macrophage membrane-coated silk fibroin nanoparticles
MSe@SiO_2_	Macrophage membrane-coated selenium-doped silica nanospheres
NF-κB	Nuclear factor kappa B
NPs	Nanoparticles
OC	Osteocalcin
OiMSCs	Osteoinductive mesenchymal stem cells
OPN	Osteopontin
PCL	Poly(ε-caprolactone)
*P. gingivalis*	*Porphyromonas gingivalis*
PLGA	Poly (lactic-co-glycolic acid)
PLL	Poly-L-lysine
ROS	Reactive oxygen species
RvD1	Resolvin D1
RUNX2	Runt-related transcription factor 2
β-TCP	Beta-tricalcium phosphate
TGF-β	Transforming growth factor beta
TLR4	Toll-like receptor 4
TNF-α	Tumour necrosis factor alpha
VCAM-1	Vascular cell adhesion molecule-1
VEGF	Vascular endothelial growth factor

## References

[1] Mata R., Yao Y., Cao W., Ding J., Zhou T., Zhai Z., Gao C. (2021). The Dynamic Inflammatory Tissue Microenvironment: Signality and Disease Therapy by Biomaterials. Research.

[2] Crupi A., Costa A., Tarnok A., Melzer S., Teodori L. (2015). Inflammation in tissue engineering: The Janus between engraftment and rejection. Eur. J. Immunol..

[3] Kumar M., Yip L., Wang F., Marty S.E., Fathman C.G. (2025). Autoimmune disease: Genetic susceptibility, environmental triggers, and immune dysregulation. Where can we develop therapies?. Front. Immunol..

[4] Yasmeen F., Pirzada R.H., Ahmad B., Choi B., Choi S. (2024). Understanding Autoimmunity: Mechanisms, Predisposing Factors, and Cytokine Therapies. Int. J. Mol. Sci..

[5] Mundhara N., Sadhukhan P. (2024). Cracking the Codes behind Cancer Cells’ Immune Evasion. Int. J. Mol. Sci..

[6] Huang M., Wang C., Li P., Lu H., Li A., Xu S. (2024). Role of immune dysregulation in peri-implantitis. Front. Immunol..

[7] Goodman S.B., Gibon E., Gallo J., Takagi M. (2022). Macrophage Polarization and the Osteoimmunology of Periprosthetic Osteolysis. Curr. Osteoporos. Rep..

[8] Zhang Z., Sui Q., Fu W., Dong X., Liu X., He Y., Zang Y., Wang W., Kou N., Liu H. (2026). Enhancing the Osseointegration of Titanium Implants by Modulating M1/M2 Macrophage Polarization through PI3K-Akt Signal Pathway on a “Cortex-like” Microarc Oxidation Coating. ACS Appl. Mater. Interfaces.

[9] Gangadhar L., Subburaj S. (2025). Nanotechnology advances for biomedical applications. Front. Nanotechnol..

[10] Alimohammadvand S., Kaveh Zenjanab M., Mashinchian M., Shayegh J., Jahanban-Esfahlan R. (2024). Recent advances in biomimetic cell membrane–camouflaged nanoparticles for cancer therapy. Biomed. Pharmacother..

[11] Ai X., Hu M., Wang Z., Zhang W., Li J., Yang H., Lin J., Xing B. (2018). Recent Advances of Membrane-Cloaked Nanoplatforms for Biomedical Applications. Bioconjugate Chem..

[12] Chen H.-W., Fang Z.-S., Chen Y.-T., Chen Y.-I., Yao B.-Y., Cheng J.-Y., Chien C.-Y., Chang Y.-C., Hu C.-M.J. (2017). Targeting and Enrichment of Viral Pathogen by Cell Membrane Cloaked Magnetic Nanoparticles for Enhanced Detection. ACS Appl. Mater. Interfaces.

[13] Chen W., Zhang Q., Luk B.T., Fang R.H., Liu Y., Gao W., Zhang L. (2016). Coating nanofiber scaffolds with beta cell membrane to promote cell proliferation and function. Nanoscale.

[14] Li H., Jin K., Luo M., Wang X., Zhu X., Liu X., Jiang T., Zhang Q., Wang S., Pang Z. (2019). Size Dependency of Circulation and Biodistribution of Biomimetic Nanoparticles: Red Blood Cell Membrane-Coated Nanoparticles. Cells.

[15] Hu C.-M.J., Fang R.H., Wang K.-C., Luk B.T., Thamphiwatana S., Dehaini D., Nguyen P., Angsantikul P., Wen C.H., Kroll A.V. (2015). Nanoparticle biointerfacing by platelet membrane cloaking. Nature.

[16] Gao C., Lin Z., Jurado-Sánchez B., Lin X., Wu Z., He Q. (2016). Stem Cell Membrane-Coated Nanogels for Highly Efficient In Vivo Tumor Targeted Drug Delivery. Small.

[17] Sun H., Su J., Meng Q., Yin Q., Chen L., Gu W., Zhang P., Zhang Z., Yu H., Wang S. (2016). Cancer-Cell-Biomimetic Nanoparticles for Targeted Therapy of Homotypic Tumors. Adv. Mater..

[18] Wu G., Ji H., Guo X., Li Y., Ren T., Dong H., Liu J., Liu Y., Shi X., He B. (2020). Nanoparticle reinforced bacterial outer-membrane vesicles effectively prevent fatal infection of carbapenem-resistant Klebsiella pneumoniae. Nanomedicine.

[19] Thamphiwatana S., Angsantikul P., Escajadillo T., Zhang Q., Olson J., Luk B.T., Zhang S., Fang R.H., Gao W., Nizet V. (2017). Macrophage-like nanoparticles concurrently absorbing endotoxins and proinflammatory cytokines for sepsis management. Proc. Natl. Acad. Sci. USA.

[20] Smith T.D., Nagalla R.R., Chen E.Y., Liu W.F. (2017). Harnessing macrophage plasticity for tissue regeneration. Adv. Drug Deliv. Rev..

[21] Chen S., Qin Z., Lin X., Zhou S., Xu Y., Zhu Y. (2025). Macrophages: Emerging targets for ulcerative colitis. Front. Immunol..

[22] Luo M., Zhao F., Cheng H., Su M., Wang Y. (2024). Macrophage polarization: An important role in inflammatory diseases. Front. Immunol..

[23] Ishina I.A., Zakharova M.Y., Kurbatskaia I.N., Mamedov A.E., Belogurov A.A., Gabibov A.G. (2023). MHC Class II Presentation in Autoimmunity. Cells.

[24] Ville S., Poirier N., Blancho G., Vanhove B. (2015). Co-Stimulatory Blockade of the CD28/CD80-86/CTLA-4 Balance in Transplantation: Impact on Memory T Cells?. Front. Immunol..

[25] Hogan K.A., Chini C.C.S., Chini E.N. (2019). The Multi-faceted Ecto-enzyme CD38: Roles in Immunomodulation, Cancer, Aging, and Metabolic Diseases. Front. Immunol..

[26] Li W., Li Y., Jin X., Liao Q., Chen Z., Peng H., Zhou Y. (2022). CD38: A Significant Regulator of Macrophage Function. Front. Oncol..

[27] Tian S., Wang Y., Wan J., Yang M., Fu Z. (2024). Co-stimulators CD40-CD40L, a potential immune-therapy target for atherosclerosis: A review. Medicine.

[28] Molteni M., Gemma S., Rossetti C. (2016). The Role of Toll-Like Receptor 4 in Infectious and Noninfectious Inflammation. Mediat. Inflamm..

[29] Arango Duque G., Descoteaux A. (2014). Macrophage cytokines: Involvement in immunity and infectious diseases. Front. Immunol..

[30] Ross E.A., Devitt A., Johnson J.R. (2021). Macrophages: The Good, the Bad, and the Gluttony. Front. Immunol..

[31] Cutolo M., Campitiello R., Gotelli E., Soldano S. (2022). The Role of M1/M2 Macrophage Polarization in Rheumatoid Arthritis Synovitis. Front. Immunol..

[32] Chen Y., Zhang X. (2017). Pivotal regulators of tissue homeostasis and cancer: Macrophages. Exp. Hematol. Oncol..

[33] Chen S., Saeed A.F.U.H., Liu Q., Jiang Q., Xu H., Xiao G.G., Rao L., Duo Y. (2023). Macrophages in immunoregulation and therapeutics. Signal Transduct. Target. Ther..

[34] Viola A., Munari F., Sánchez-Rodríguez R., Scolaro T., Castegna A. (2019). The Metabolic Signature of Macrophage Responses. Front. Immunol..

[35] Xia T., Fu S., Yang R., Yang K., Lei W., Yang Y., Zhang Q., Zhao Y., Yu J., Yu L. (2023). Advances in the study of macrophage polarization in inflammatory immune skin diseases. J. Inflamm..

[36] Pourcet B., Pineda-Torra I. (2013). Transcriptional regulation of macrophage arginase 1 expression and its role in atherosclerosis. Trends Cardiovasc. Med..

[37] Karadima E., Chavakis T., Alexaki V.I. (2025). Arginine metabolism in myeloid cells in health and disease. Semin. Immunopathol..

[38] van der Zande H.J.P., Nitsche D., Schlautmann L., Guigas B., Burgdorf S. (2021). The Mannose Receptor: From Endocytic Receptor and Biomarker to Regulator of (Meta)Inflammation. Front. Immunol..

[39] Cummings R.D. (2022). The mannose receptor ligands and the macrophage glycome. Curr. Opin. Struct. Biol..

[40] Etzerodt A., Moestrup S.K. (2013). CD163 and inflammation: Biological, diagnostic, and therapeutic aspects. Antioxid. Redox Signal..

[41] Moestrup S.K., Møller H.J. (2004). CD163: A regulated hemoglobin scavenger receptor with a role in the anti-inflammatory response. Ann. Med..

[42] Kelley J.L., Ozment T.R., Li C., Schweitzer J.B., Williams D.L. (2014). Scavenger receptor-A (CD204): A two-edged sword in health and disease. Crit. Rev. Immunol..

[43] Gudgeon J., Marín-Rubio J.L., Trost M. (2022). The role of macrophage scavenger receptor 1 (MSR1) in inflammatory disorders and cancer. Front. Immunol..

[44] Mariani E., Lisignoli G., Borzì R.M., Pulsatelli L. (2019). Biomaterials: Foreign Bodies or Tuners for the Immune Response?. Int. J. Mol. Sci..

[45] Rahnama-Hezavah M., Mertowska P., Mertowski S., Skiba J., Krawiec K., Łobacz M., Grywalska E. (2023). How Can Imbalance in Oral Microbiota and Immune Response Lead to Dental Implant Problems?. Int. J. Mol. Sci..

[46] Au A.E., Josefsson E.C. (2017). Regulation of platelet membrane protein shedding in health and disease. Platelets.

[47] Mason K.D., Carpinelli M.R., Fletcher J.I., Collinge J.E., Hilton A.A., Ellis S., Kelly P.N., Ekert P.G., Metcalf D., Roberts A.W. (2007). Programmed anuclear cell death delimits platelet life span. Cell.

[48] Chen Z., Klein T., Murray R.Z., Crawford R., Chang J., Wu C., Xiao Y. (2016). Osteoimmunomodulation for the development of advanced bone biomaterials. Mater. Today.

[49] Rodríguez-Morales P., Franklin R.A. (2023). Macrophage phenotypes and functions: Resolving inflammation and restoring homeostasis. Trends Immunol..

[50] Gao M., Guo H., Dong X., Wang Z., Yang Z., Shang Q., Wang Q. (2024). Regulation of inflammation during wound healing: The function of mesenchymal stem cells and strategies for therapeutic enhancement. Front. Pharmacol..

[51] Yu Y., Wu H., Zhang Q., Ogawa R., Fu S. (2021). Emerging insights into the immunological aspects of keloids. J. Dermatol..

[52] King A., Balaji S., Le L.D., Crombleholme T.M., Keswani S.G. (2014). Regenerative Wound Healing: The Role of Interleukin-10. Adv. Wound Care.

[53] Bohara S., Suthakorn J. (2022). Surface coating of orthopedic implant to enhance the osseointegration and reduction of bacterial colonization: A review. Biomater. Res..

[54] Gao W., Xiao Y. (2022). Advances in cell membrane-encapsulated biomaterials for tissue repair and regeneration. Appl. Mater. Today.

[55] Wang C., Zhao Q., Chen C., Li J., Zhang J., Qu S., Tang H., Zeng H., Zhang Y. (2023). CD301b^+^ macrophage: The new booster for activating bone regeneration in periodontitis treatment. Int. J. Oral Sci..

[56] Parodi A., Quattrocchi N., Van De Ven A.L., Chiappini C., Evangelopoulos M., Martinez J.O., Brown B.S., Khaled S.Z., Yazdi I.K., Enzo M.V. (2013). Synthetic nanoparticles functionalized with biomimetic leukocyte membranes possess cell-like functions. Nat. Nanotechnol..

[57] Li S., Wang L., Gu Y., Lin L., Zhang M., Jin M., Mao C., Zhou J., Zhang W., Huang X. (2021). Biomimetic immunomodulation by crosstalk with nanoparticulate regulatory T cells. Matter.

[58] Pan S., Zhong W., Lan Y., Yu S., Yang L., Yang F., Li J., Gao X., Song J. (2024). Pathology-Guided Cell Membrane-Coated Polydopamine Nanoparticles for Efficient Multisynergistic Treatment of Periodontitis. Adv. Funct. Mater..

[59] Wang H., Wu J., Williams G.R., Fan Q., Niu S., Wu J., Xie X., Zhu L.M. (2019). Platelet-membrane-biomimetic nanoparticles for targeted antitumor drug delivery. J. Nanobiotechnol..

[60] Fang R.H., Kroll A.V., Gao W., Zhang L. (2018). Cell Membrane Coating Nanotechnology. Adv. Mater..

[61] Zhao C., Song W., Ma J., Wang N. (2022). Macrophage-derived hybrid exosome-mimic nanovesicles loaded with black phosphorus for multimodal rheumatoid arthritis therapy. Biomater. Sci..

[62] Nakkala J.R., Duan Y., Ding J., Muhammad W., Zhang D., Mao Z., Ouyang H., Gao C. (2022). Macrophage membrane-functionalized nanofibrous mats and their immunomodulatory effects on macrophage polarization. Acta Biomater..

[63] Teo K.Y.W., Sevencan C., Cheow Y.A., Zhang S., Leong D., Toh W.S. (2022). Macrophage Polarization as a Facile Strategy to Enhance Efficacy of Macrophage Membrane-Coated Nanoparticles in Osteoarthritis. Small Sci..

[64] Zhang Z., Li D., Li X., Guo Z., Liu Y., Ma X., Zheng S. (2020). PEI-modified macrophage cell membrane-coated PLGA nanoparticles encapsulating Dendrobium polysaccharides as a vaccine delivery system for ovalbumin to improve immune responses. Int. J. Biol. Macromol..

[65] Gao C., Chen Y., Cheng X., Zhang Y., Zhang Y., Wang Y., Cui Z., Liao Y., Luo P., Wu W. (2022). A novel structurally identified epitope delivered by macrophage membrane-coated PLGA nanoparticles elicits protection against Pseudomonas aeruginosa. J. Nanobiotechnol..

[66] Wen X., Xiong X., Yang G., Xiao W., Hou J., Pan T., Hu Y., Zhou S. (2023). A macrophage membrane-coated mesoporous silica nanoplatform inhibiting adenosine A2AR via in situ oxygen supply for immunotherapy. J. Control. Release.

[67] Meng Q.F., Rao L., Zan M., Chen M., Yu G.T., Wei X., Wu Z., Sun Y., Guo S.S., Zhao X.Z. (2018). Macrophage membrane-coated iron oxide nanoparticles for enhanced photothermal tumor therapy. Nanotechnology.

[68] Xiao T., He M., Xu F., Fan Y., Jia B., Shen M., Wang H., Shi X. (2021). Macrophage Membrane-Camouflaged Responsive Polymer Nanogels Enable Magnetic Resonance Imaging-Guided Chemotherapy/Chemodynamic Therapy of Orthotopic Glioma. ACS Nano.

[69] Quijia C.R., Navegante G., Sábio R.M., Valente V., Ocaña A., Alonso-Moreno C., Frem R.C., Chorilli M. (2023). Macrophage Cell Membrane Coating on Piperine-Loaded MIL-100(Fe) Nanoparticles for Breast Cancer Treatment. J. Funct. Biomater..

[70] Xuan M., Shao J., Dai L., He Q., Li J. (2015). Macrophage Cell Membrane Camouflaged Mesoporous Silica Nanocapsules for In Vivo Cancer Therapy. Adv. Healthc. Mater..

[71] Cao H., Dan Z., He X., Zhang Z., Yu H., Yin Q., Li Y. (2016). Liposomes Coated with Isolated Macrophage Membrane Can Target Lung Metastasis of Breast Cancer. ACS Nano.

[72] Fernández-Borbolla A., García-Hevia L., Fanarraga M.L. (2024). Cell Membrane-Coated Nanoparticles for Precision Medicine: A Comprehensive Review of Coating Techniques for Tissue-Specific Therapeutics. Int. J. Mol. Sci..

[73] Khatoon N., Zhang Z., Zhou C., Chu M. (2022). Macrophage membrane coated nanoparticles: A biomimetic approach for enhanced and targeted delivery. Biomater. Sci..

[74] Yi X., Gao H. (2014). Phase diagrams and morphological evolution in wrapping of rod-shaped elastic nanoparticles by cell membrane: A two-dimensional study. Phys. Rev. E.

[75] Kroll A.V., Fang R.H., Zhang L. (2017). Biointerfacing and Applications of Cell Membrane-Coated Nanoparticles. Bioconjugate Chem..

[76] Wei X., Gao J., Fang R.H., Luk B.T., Kroll A.V., Dehaini D., Zhou J., Kim H.W., Gao W., Lu W. (2016). Nanoparticles camouflaged in platelet membrane coating as an antibody decoy for the treatment of immune thrombocytopenia. Biomaterials.

[77] Liu L., Bai X., Martikainen M.V., Kårlund A., Roponen M., Xu W., Hu G., Tasciotti E., Lehto V.P. (2021). Cell membrane coating integrity affects the internalization mechanism of biomimetic nanoparticles. Nat. Commun..

[78] Molinaro R., Corbo C., Martinez J.O., Taraballi F., Evangelopoulos M., Minardi S., Yazdi I.K., Zhao P., De Rosa E., Sherman M.B. (2016). Biomimetic proteolipid vesicles for targeting inflamed tissues. Nat. Mater..

[79] Molinaro R., Evangelopoulos M., Hoffman J.R., Corbo C., Taraballi F., Martinez J.O., Hartman K.A., Cosco D., Costa G., Romeo I. (2018). Design and Development of Biomimetic Nanovesicles Using a Microfluidic Approach. Adv. Mater..

[80] Luk B.T., Hu C.M., Fang R.H., Dehaini D., Carpenter C., Gao W., Zhang L. (2014). Interfacial interactions between natural RBC membranes and synthetic polymeric nanoparticles. Nanoscale.

[81] Liu L., Wang Y., Guo X., Zhao J., Zhou S. (2020). A Biomimetic Polymer Magnetic Nanocarrier Polarizing Tumor-Associated Macrophages for Potentiating Immunotherapy. Small.

[82] Wu Y., Wan S., Yang S., Hu H., Zhang C., Lai J., Zhou J., Chen W., Tang X., Luo J. (2022). Macrophage cell membrane-based nanoparticles: A new promising biomimetic platform for targeted delivery and treatment. J. Nanobiotechnol..

[83] Wei Y., Zhu M., Li S., Hong T., Guo X., Li Y., Liu Y., Hou X., He B. (2021). Engineered Biomimetic Nanoplatform Protects the Myocardium Against Ischemia/Reperfusion Injury by Inhibiting Pyroptosis. ACS Appl. Mater. Interfaces.

[84] Wang Y., Zhang D., Jia M., Zheng X., Liu Y., Wang C., Lei F., Niu H., Li C. (2022). ZIF-8 nanoparticles coated with macrophage-derived microvesicles for sustained, targeted delivery of dexamethasone to arthritic joints. J. Drug Target..

[85] Yang F., Cabe M.H., Ogle S.D., Sanchez V., Langert K.A. (2021). Optimization of critical parameters for coating of polymeric nanoparticles with plasma membrane vesicles by sonication. Sci. Rep..

[86] Yu M., Xu L., Tian F., Su Q., Zheng N., Yang Y., Wang J., Wang A., Zhu C., Guo S. (2018). Rapid transport of deformation-tuned nanoparticles across biological hydrogels and cellular barriers. Nat. Commun..

[87] Kong S.M., Costa D.F., Jagielska A., Van Vliet K.J., Hammond P.T. (2021). Stiffness of targeted layer-by-layer nanoparticles impacts elimination half-life, tumor accumulation, and tumor penetration. Proc. Natl. Acad. Sci. USA.

[88] Liang Q., Bie N., Yong T., Tang K., Shi X., Wei Z., Jia H., Zhang X., Zhao H., Huang W. (2019). The softness of tumour-cell-derived microparticles regulates their drug-delivery efficiency. Nat. Biomed. Eng..

[89] Zou D., Wu Z., Yi X., Hui Y., Yang G., Liu Y., Tengjisi, Wang H., Brooks A., Wang H. (2023). Nanoparticle elasticity regulates the formation of cell membrane-coated nanoparticles and their nano-bio interactions. Proc. Natl. Acad. Sci. USA.

[90] Hou Y., Wu R., Zhou Y., Yin C., Gai Y., Jiang D., Wang K., Xia X. (2025). Macrophage membrane-coated nanoparticles in inflammatory diseases: From bioinspired design to translational potential. J. Nanobiotechnol..

[91] Zhao X., Chen W., Wu J., Shen Y., Xu B., Chen Z., Sun Y. (2025). Application of Biomimetic Cell Membrane-Coated Nanocarriers in Cardiovascular Diseases. Int. J. Nanomed..

[92] Chou L.Y., Ming K., Chan W.C. (2011). Strategies for the intracellular delivery of nanoparticles. Chem. Soc. Rev..

[93] Shi M., Shen K., Yang B., Zhang P., Lv K., Qi H., Wang Y., Li M., Yuan Q., Zhang Y. (2021). An electroporation strategy to synthesize the membrane-coated nanoparticles for enhanced anti-inflammation therapy in bone infection. Theranostics.

[94] Yuan S., Hu D., Gao D., Butch C.J., Wang Y., Zheng H., Sheng Z. (2025). Recent advances of engineering cell membranes for nanomedicine delivery across the blood–brain barrier. J. Nanobiotechnol..

[95] Fang R.H., Gao W., Zhang L. (2023). Targeting drugs to tumours using cell membrane-coated nanoparticles. Nat. Rev. Clin. Oncol..

[96] Zeng Y., Li S., Zhang S., Wang L., Yuan H., Hu F. (2022). Cell membrane coated-nanoparticles for cancer immunotherapy. Acta Pharm. Sin. B.

[97] Zhao X., Xu Z., Wang D., Li T., Xu L., Li Z., Bai X., Zhu H., Liu Y., Wang Y. (2025). Nanotechnology-based targeted regulation of NLRP3 Inflammasome: Therapeutic strategies and clinical application prospects in inflammatory diseases. Drug Deliv..

[98] Ding C., Yang C., Cheng T., Wang X., Wang Q., He R., Sang S., Zhu K., Xu D., Wang J. (2021). Macrophage-biomimetic porous Se@SiO2 nanocomposites for dual modal immunotherapy against inflammatory osteolysis. J. Nanobiotechnol..

[99] Qiao F., Lv Y. (2023). Hybrid Cell Membrane-Functionalized Matrixes for Modulating Inflammatory Microenvironment and Improving Bone Defect Repair. Adv. Healthc. Mater..

[100] Ma Y., Yang H., Zong X., Wu J., Ji X., Liu W., Yuan P., Chen X., Yang C., Li X. (2021). Artificial M2 macrophages for disease-modifying osteoarthritis therapeutics. Biomaterials.

[101] Zhou K., Yang C., Shi K., Liu Y., Hu D., He X., Yang Y., Chu B., Peng J., Zhou Z. (2023). Activated macrophage membrane-coated nanoparticles relieve osteoarthritis-induced synovitis and joint damage. Biomaterials.

[102] Yin C., Zhao Q., Li W., Zhao Z., Wang J., Deng T., Zhang P., Shen K., Li Z., Zhang Y. (2020). Biomimetic anti-inflammatory nano-capsule serves as a cytokine blocker and M2 polarization inducer for bone tissue repair. Acta Biomater..

[103] Lasserre J.F., Brecx M.C., Toma S. (2018). Oral Microbes, Biofilms and Their Role in Periodontal and Peri-Implant Diseases. Materials.

[104] Shuyu G., Gu J., Jiang Y., Cui W., Chen J., Li L., Zheng K., Xu Y. (2022). Pretreatment of macrophage-membrane-coated nanoparticles for therapeutical targeting of *P. gingivalis*-accelerated atherosclerosis. Mater. Des..

[105] Deng Y., Ren M., He P., Liu F., Wang X., Zhou C., Li Y., Yang S. (2023). Genetically engineered cell membrane-coated nanoparticles for antibacterial and immunoregulatory dual-function treatment of ligature-induced periodontitis. Front. Bioeng. Biotechnol..

[106] Song R., Wan Z., Yuan X., Wang N., Gao Y., Zhang L., Ren H., Jin Y., Liu X., Sang J. (2025). Macrophage membrane functionalized composite microspheres promote bone regeneration in periodontitis via manipulating inflammation reversing-osteogenesis coupling. Mater. Today Bio.

[107] Saadh M.J., Saeed T.N., Alfarttoosi K.H., Sanghvi G., Roopashree R., Thakur V., Lakshmi L., Kubaev A., Taher W.M., Alwan M. (2025). Exosomes and MicroRNAs: Key modulators of macrophage polarization in sepsis pathophysiology. Eur. J. Med. Res..

[108] Kafle U., Thapa R., Panth N., Suwal N., Bashyal S., Bhatia R., Arora M., Chellappan D.K., Gupta G., Gulati M. (2026). ROS-responsive drug delivery systems: Harnessing redox biology for targeted therapies. Colloids Surf. B Biointerfaces.

[109] Chen X., Zhan Y., Fan X., Pan J., lv Y., Wang W., Jiang W., Liu Y., Tang J. (2026). Nanozymes for inflammatory disease therapy: ROS homeostasis and clinical prospects. Chem. Eng. J..

[110] Gasmi Benahmed A., Gasmi A., Tippairote T., Mujawdiya P.K., Avdeev O., Shanaida Y., Bjørklund G. (2022). Metabolic Conditions and Peri-Implantitis. Antibiotics.

[111] Yang S., Yang X. (2025). The Role of Reactive Oxygen Species (ROS) in Periodontitis: A Potential Therapeutic Target. Immun. Inflamm. Dis..

[112] Zhang H., Han S., Zhang X., Ma J., Jin L., Wang Y., Ma Y., Ma T., Yu F., Song G. (2026). Novel Lipid Nanoparticle (LNP) Delivery Systems Enabling the Advancement of RNA Therapeutics. Adv. Healthc. Mater..

[113] Sun L., Wang J., Song Q., Li F., Ling D., Hu X. (2026). Advances in artificial metabzymes for macrophage polarization in tumor metabolic immunotherapy. Mater. Today Bio.

[114] Ding Y., Liu G., Liu S., Li X., Xu K., Liu P., Cai K. (2023). A Multifunction Hydrogel-Coating Engineered Implant for Rescuing Biofilm Infection and Boosting Osseointegration by Macrophage-Related Immunomodulation. Adv. Healthc. Mater..

[115] Ding Y., Liu T. (2025). MACROPHAGE MEMBRANE-COATED NANOPARTICLES DELIVERING ANTAGOMIR OF MIR-155-5P MITIGATED TITANIUM PARTICLE-INDUCED OSTEOLYSIS BY MODULATING PERIPROSTHETIC MICROENVIRONMENT VIA GAS6/AXL SIGNALLING. Orthop. Proc..

[116] Wu J., Chen M., Xiao Y., Yang H., Wang G., Zhang X., Dai L., Yuan Z. (2024). The Bioactive Interface of Titanium Implant with Both Anti-Oxidative Stress and Immunomodulatory Properties for Enhancing Osseointegration under Diabetic Condition. Adv. Healthc. Mater..

[117] Yang Y., Wang J., Lin X., Zhang Z., Zhang M., Tang C., Kou X., Deng F. (2024). TNF-α-licensed exosome-integrated titaniumaccelerated T2D osseointegration by promoting autophagy-regulated M2 macrophage polarization. Biochem. Biophys. Res. Commun..

[118] Baheti W., Chen X., La M., He H. (2024). Biomimetic HA-GO implant coating for enhanced osseointegration via macrophage M2 polarization-induced osteo-immunomodulation. J. Appl. Biomater. Funct. Mater..

[119] Wu H., Chen C., Li J., Yu D., Wu X., Huang H., Tang Z., Wu Q., Yan S., Wang N. (2024). Engineered Magneto-Piezoelectric Nanoparticles-Enhanced Scaffolds Disrupt Biofilms and Activate Oxidative Phosphorylation in Icam1+ Macrophages for Infectious Bone Defect Regeneration. ACS Nano.

[120] Wang N., Zhao Q., Gong Z., Fu L., Li J., Hu L. (2022). CD301b+ Macrophages as Potential Target to Improve Orthodontic Treatment under Mild Inflammation. Cells.

[121] Qu Y., Chu B., Li J., Deng H., Niu T., Qian Z. (2024). Macrophage-Biomimetic Nanoplatform-Based Therapy for Inflammation-Associated Diseases. Small Methods.

[122] Liu J., Liu Z., Pang Y., Zhou H. (2022). The interaction between nanoparticles and immune system: Application in the treatment of inflammatory diseases. J. Nanobiotechnol..

[123] Brown B.N., Ratner B.D., Goodman S.B., Amar S., Badylak S.F. (2012). Macrophage polarization: An opportunity for improved outcomes in biomaterials and regenerative medicine. Biomaterials.

